# Interaction effects of high temperature and ozone on cardiovascular disease mortality in Chengdu, 2014–2023

**DOI:** 10.3389/fpubh.2025.1580849

**Published:** 2025-07-10

**Authors:** Jinqiu Yao, Jingwen Sun, Dan Kuang, Wen Qian, Li Luo, Fangkui Qin, Yifan Zhai, Yueling Li, Jiaqi Huang, Cheng Wang, Rong Lu, XuFang Gao

**Affiliations:** Chengdu Center for Disease Control and Prevention, Chengdu, China

**Keywords:** ozone (O_3_), high temperature, interaction, cardiovascular mortality, distributed lag non-linear model

## Abstract

**Background:**

Ozone pollution has significantly worsened in recent years. In the context of global warming, the frequency and intensity of heat waves have significantly increased. Ozone levels exhibit a significant dose–response relationship with cardiovascular disease risk, and high temperatures have been demonstrated to be one of the critical risk factors for cardiovascular disease. Notably, these two hazards potentially create synergistic health impacts through combined exposure effects.

**Objectives:**

We aimed to estimate the interaction effects between high temperature, ozone and the death of multiple cardiovascular diseases.

**Methods:**

We analyzed daily mortality records for cardiovascular disease types alongside meteorological and environmental parameters in Chengdu (2014–2023) through a two-stage analytical framework. In the first stage, we explored the relations between ozone exposure and multiple cardiovascular diseases, then the Distributed Lag Non-Linear Model (DLNM) was utilized to capture the lagged and cumulative effects of temperature and ozone levels on the death of different cardiovascular disease types. In the second stage, both quantitative and qualitative analyses were conducted to further explore their dose–response relationships. Additionally, stratification analyses on gender, age, education level and marital status were also performed.

**Results:**

We observed positive correlations between ozone levels and the death of cardiovascular disease, cerebrovascular disease, coronary heart disease, cerebral infarction, heart attack, hypertension and stroke. The highest cumulative lag effect observed was 3 days. Furthermore, the associations were stronger in women, the older adult, individuals with lower education levels, and unmarried people. High temperature and elevated ozone levels synergistically increased the mortality risk, and the relative excess risk to interaction (RERI, 95% CI) values were cardiovascular disease 0.201 (0.149–0.268), cerebrovascular disease 0.177 (0.099–0.255), coronary heart disease 0.281 (0.169–0.394), cerebral infarction 0.582 (0.369–0.794), stroke 0.287 (0.135–0.441), and 0.482 (0.269–0.694), respectively.

**Conclusion:**

We observed the synergistic interactions between ozone levels and high temperature on the death of cardiovascular disease, cerebrovascular disease, coronary heart disease, cerebral infarction, heart attack and stroke. The associations were stronger in women, the older adult, individuals with lower education levels, and unmarried people.

## 1 Introduction

Ozone pollution has emerged as a critical global health challenge, with an increased risk to human health in recent years (1). Epidemiological evidence confirms a dose-dependent relationship between ozone exposure and cardiovascular mortality, with every 10 ppb increase in maximum daily 8-h average ozone concentration corresponding to a 31% elevation in cardiovascular disease mortality risk (2). The 2021 Global Burden of Disease report attributes approximately 489,000 annual deaths and 8.77 million disability-adjusted life years lost worldwide to ambient ozone pollution (3), underscoring its substantial disease burden. Given its severe effects on disease burden and increasing trend, ozone pollution has become a major health challenge globally (4).

Concurrently, accelerating climate change has amplified heatwave frequency. The Lancet Countdown China report indicates that heatwave exposure days per capita increased by 4.51 days in 2020 compared to the average level from 1986 to 2005 (5, 6). Exposure to high temperature affects human health. During 1990 and 2019, high temperature cause 11.7 million years of healthy life lost globally (7). Thermal stress induces pathophysiological changes, including blood hyperviscosity and prothrombotic states, while laboratory models demonstrate direct cardiotoxicity through cardiomyocytes, which increased vacuolisation and cardiac mitochondrial remodeling (8). As a critical risk factor contributing to cardiovascular disease, further studies on the effects of high temperature are essential.

Ozone pollution and high temperature may coincide as the same formation condition, making worse health effects (9). The synergistic interactions between ozone, temperature, and the death of cardiovascular diseases need more attention. Current research predominantly originate from developed countries, with studies from Europe (10–12) and the United States (US) (9), demonstrating ozone-temperature interactions on cardiovascular mortality. Of note, these interactions have so far only been demonstrated to exist under specific conditions, studies in European countries have only observed significant associations during the warm season (10, 13). Emerging evidence from developing countries also reveals spatial heterogeneity in ozone-temperature health effects. Studies carried out in Shijiazhuang (14) demonstrate that when O_3_ ≥ 100 μg/m^3^ and T ≥ 27.5°C, every 1°C increase in temperature was related with 6.8% (95% CI:1.025, 1.114) increase in the circulatory system. Two studies conducted in Jiangsu (15) and Nanjing (16) demonstrated that higher temperature and ozone concentrations can increase human ischemic heart disease (IHD) mortality. While the study in Shenzhen (17) found that ozone decrease the risk of hypertensive diseases morality in warm season.

As the largest southwestern China metropolis occupying the central Sichuan Basin, Chengdu’s unique topography promotes atmospheric stagnation that exacerbates pollutant accumulation (18). Most previous studies focus on overall or specific cardiovascular disease, and their results have been inconsistent. Existing studies (9–16, 19–21) show inconsistent conclusions regarding single-exposure effects, crucially lacking investigation into the synergistic associations between high temperature and ozone exposure on the death of different types of cardiovascular diseases. Therefore, given the basin’s distinctive air pollution meteorology, studies conducted in the Sichuan Basin are particularly important. Our study provides valuable insights for public health. Furthermore, it offers substantial epidemiological evidence for policymakers in southwestern China to enhance strategies for controlling ozone pollution.

## 2 Materials and methods

### 2.1 Study population

We obtained daily cardiovascular diseases mortality records from 1 January, 2014 to 31 December, 2023 through the Death Surveillance System of China Center for Disease Control and Prevention, with analysis restricted to summer months (May to September) across 2014–2023. We included the permanent residents, non-Chengdu residents were excluded based on the information of home address verification. Cardiovascular diseases (codes: I00-I99) were categorized according to the International Classification of Diseases, 10th edition (ICD-10), which include cardiovascular disease (I00-I99), cerebrovascular disease (I60-I69), coronary heart disease (I20-I25), hypertension (I10-I15), rheumatic heart disease (I00-I02, I05-I09), cerebral hemorrhage (I61), stroke (I64), cerebral infarction (I63) and heart attack (I21).

### 2.2 Meteorological and air pollutants data

The meteorological data were obtained from Chengdu Meteorological Bureau, including daily average temperature (°C), relative humidity (%) and daily average pressure (Pa), etc. The daily concentrations of air pollutants in Chengdu were collected from Chengdu Environmental Monitoring Center, including PM_2.5_ (24-h mean, μg/m^3^) and O_3_ (8-h maximum, μg/m^3^). All data underwent strict quality control. Missing values were filled in with the average of the three neighboring values to maintain temporal continuity across meteorological and environmental variables.

### 2.3 Statistical analysis

To avoid the influence of co-linearity between meteorology and air pollutants on the subsequent analysis, spearman’s correlation coefficient (SCC) was used to explore relations between meteorological factors and environmental data. Afterwards, the statistical analysis were carried in two stages.

In the first stage, we explored associations between ozone exposure and different types of cardiovascular diseases. Then we used the Distributed Lag Non-Linear Model (DLNM) to capture the lagged and cumulative effects of high temperature and ozone levels on the death of different types of cardiovascular diseases. The equation is as follows:


$$\begin{array}{l} \text{LogE}(Y_{t})=\alpha +\beta^{*}(X_{t})+\mathrm{ns}(\text{times},\mathrm{df})+\mathrm{ns}(\text{humidity},\mathrm{df})\\ +\mathrm{ns}(\text{pressure},\mathrm{df})+\mathrm{ns}(NO_{2},\mathrm{df})+\mathrm{ns}(PM_{2.5},\mathrm{df})\\ +\mathrm{Dow}+\text{Holiday} \end{array}$$

E(Y_t_) is the expected number of deaths from different types of cardiovascular diseases on day *t*, *α* is fit constant, *β* is the regression coefficient, where X_t_ represents the daily 8-h maximum ozone concentrations, and df is the degree of freedom, Dow is an indicator of what day of the week, Holiday is the holiday effect.

In the second stage, the quantitative and qualitative analysis were employed to explore their dose–response relations between ozone, temperature, and the death of different cardiovascular diseases. The 3D graph was further constructed to explore their interactions. To quantitatively evaluate the effects of the interactions between high temperature and ozone levels on the cardiovascular mortality, the median value was selected as the cut-off point and included in the model, characterized through the following formula:


$$\begin{array}{l} \text{LogE}(Y_{t})=\alpha +\mathrm{ns}(\text{ozone}:\text{temperature})\\ +\mathrm{ns}(\text{times},\mathrm{df})+\mathrm{ns}(\text{humidity},\mathrm{df})+\mathrm{ns}(\text{pressure},\mathrm{df})\\ +\mathrm{ns}(NO_{2},\mathrm{df})+\mathrm{ns}(PM_{2.5},\mathrm{df})+\mathrm{Dow}+\text{Holiday} \end{array}$$

ns(ozone:temperature): the interaction between ozone levels on the day of the strongest lag effects and temperature. The relative excess risk to interaction (RERI) and attributable proportion (API) were applied to evaluate the interaction.


$$\text{RERI}=R_{11}-R_{10}+R_{00}$$

$$\mathrm{API}=\text{RERI}/RR_{11}$$

R_11_ indicates the presence of both exposure factors, R_01_ and R_10_ indicate the presence of only one exposure factor, and R_00_ indicates the absence of exposure factors, set as 1. When RERI>0 and API > 0, there are synergistic effects; when RERI<0 and API < 0, there are antagonistic effects.

In addition to the main analysis described above, we conducted a stratified analysis of the study population by gender, age, education level, and marital status. All results are expressed as relative risk (RR) and 95% confidence interval (95% CI), with *p* < 0.05 as the test of statistical significance. For all analyses, we employed the “dlnm” and “mgcv” packages in R software (version 4.3.2). A two-tailed t-test was used in all statistical analyses, and *p* < 0.05 was deemed statistically significant.

Sensitivity analysis was used to test the robustness. The freedom of times and other confounders were explored based on the Akaike Information Criterion (AIC) and shoen in the Supplementary material, and our results were the same as previous studies (22, 23). In our analysis, 0–10 days was chosen as a lag range, ns with 7 freedom was used to control the long-term trends and seasonal fluctuations, and the freedom of other confounders were 3.

## 3 Results

### 3.1 Descriptive statistics

Table 1 summarizes the distribution of 9 types of cardiovascular diseases, meteorological factors, and air pollutants during the survey period. The death counts ranged from 34 to 269 in cardiovascular disease, 15 to 131 in cerebrovascular disease, 6 to 99 in coronary heart disease, 0 to 25 in hypertension, 0 to 7 in rheumatic heart disease, 2 to 36 in cerebral hemorrhage, 0 to 14 in stroke, 1 to 48 in cerebral infarction, and 1 to 57 in heart attack. The average temperature in Chengdu was 23.96 ± 3°C, the average RH was 79.18 ± 9.52%, and the average ozone level was 126.69 ug/m^3^.

**Table 1:** Summary statistics of the meteorological factors and environmental data in Chengdu between 2014 and 2023.

Variables		Average	Standard deviation	Min	P25	P50	P75	Max
Deaths from circulatory diseases	Cardiovascular disease	67.05	20.08	34.00	56.00	64.00	74.00	269.00
	Cerebrovascular disease	35.21	10.27	15.00	29.00	34.00	40.00	131.00
	Coronary heart disease	22.85	8.97	6.00	17.00	22.00	26.00	99.00
	Hypertension	4.79	2.64	0.00	3.00	5.00	6.00	25.00
	Rheumatic heart disease	1.22	1.12	0.00	0.00	1.00	2.00	7.00
	Cerebral hemorrhage	14.31	4.69	2.00	11.00	14.00	17.00	36.00
	Stroke	1.47	1.40	0.00	0.00	1.00	2.00	14.00
	Cerebral infarction	10.64	4.97	1.00	7.00	10.00	13.00	48.00
	Heart attack	12.26	5.24	1.00	9.00	12.00	15.00	57.00
Meteorological data	T/°C	23.96	3.00	15.00	21.80	24.05	26.15	33.00
	P/(Pa)	943.41	4.54	933.00	940.10	942.80	946.40	970.00
	RH/%	79.18	9.52	37.00	73.61	80.00	86.30	99.00
Air pollutants data	CO/(μg/m^3^)	0.72	0.17	0.00	0.60	0.70	0.81	2.00
	NO2/(μg/m^3^)	33.52	12.84	1.00	24.00	32.00	41.89	83.00
	SO2/(μg/m^3^)	8.80	5.33	2.00	4.70	7.65	11.78	35.00
	O_3_/(μg/m^3^)	126.69	47.93	27.00	87.00	124.35	161.94	305.00
	PM_2.5_/(μg/m^3^)	33.25	18.15	4.00	20.32	29.50	42.71	144.00
	PM10/(μg/m^3^)	55.55	29.87	5.00	34.00	49.04	70.04	210.00

The concentration of ozone varies periodically (Supplementary Figure S1), peaking during summer months and reaching its trough in winter, with temperature showing synchronized seasonal variability. Spearman’s correlation coefficient identified potential confounding factors with ozone (Supplementary Table S1): PM_2.5_ (*r* = −0.31, *p* < 0.05), NO_2_ (*r* = 0.28, *p* < 0.05), SO_2_ (*r* = 0.15, *p* < 0.05), pressure (*r* = −0.31, *p* < 0.05), temperature (*r* = 0.32, *p* < 0.05), humidity (*r* = −0.39, *p* < 0.05).

### 3.2 The character of cardiovascular diseases death counts in subgroup analysis

Table 2 shows general characteristic of the deaths of cardiovascular diseases in stratification analysis. Our study included 352,400 subjects aged 0–99 years, of whom 40.9% were women. The daily average number of overall cardiovascular disease deaths was 31 among females and 36 among males. Additionally, 36.4% of the population was over 80 years old, with a daily death count of 31 due to cardiovascular diseases. Married individuals comprised 63.2% of the population, with the daily death counts of 38. The percentage of individuals with a junior high school education or higher was 13.1%, with a corresponding daily death number of 7.

**Table 2:** Basic characteristics of the daily number of overall cardiovascular disease in different groups.

Cardiovascular diseases death counts	Sum	Mean±SD	P50 (P25, P75)	Min, Max
Total	352,400			
Gender				
Males	207,939	35.76 ± 10.72	34 (29,41)	15, 141
Females	144,461	31.29 ± 11.11	30 (25, 35)	12, 150
Age				
age>80	128,320	30.97 ± 11.57	29 (24, 36)	10, 147
age≤80	224,080	36.07 ± 10.21	35 (30, 41)	17, 129
Marital status				
Married	222,623	38.27 ± 10.49	37 (32, 43)	17, 123
others	129,777	28.78 ± 11.35	27 (22, 33)	8, 148
Education levels				
Junior high school and above	45,916	7.39 ± 3.46	7 (5, 9)	0, 29
others	306,484	59.66 ± 18.71	57 (50, 65)	28, 245

### 3.3 The exposure-response relations between high temperature, ozone and different types of cardiovascular diseases deaths

**Figure 1:**
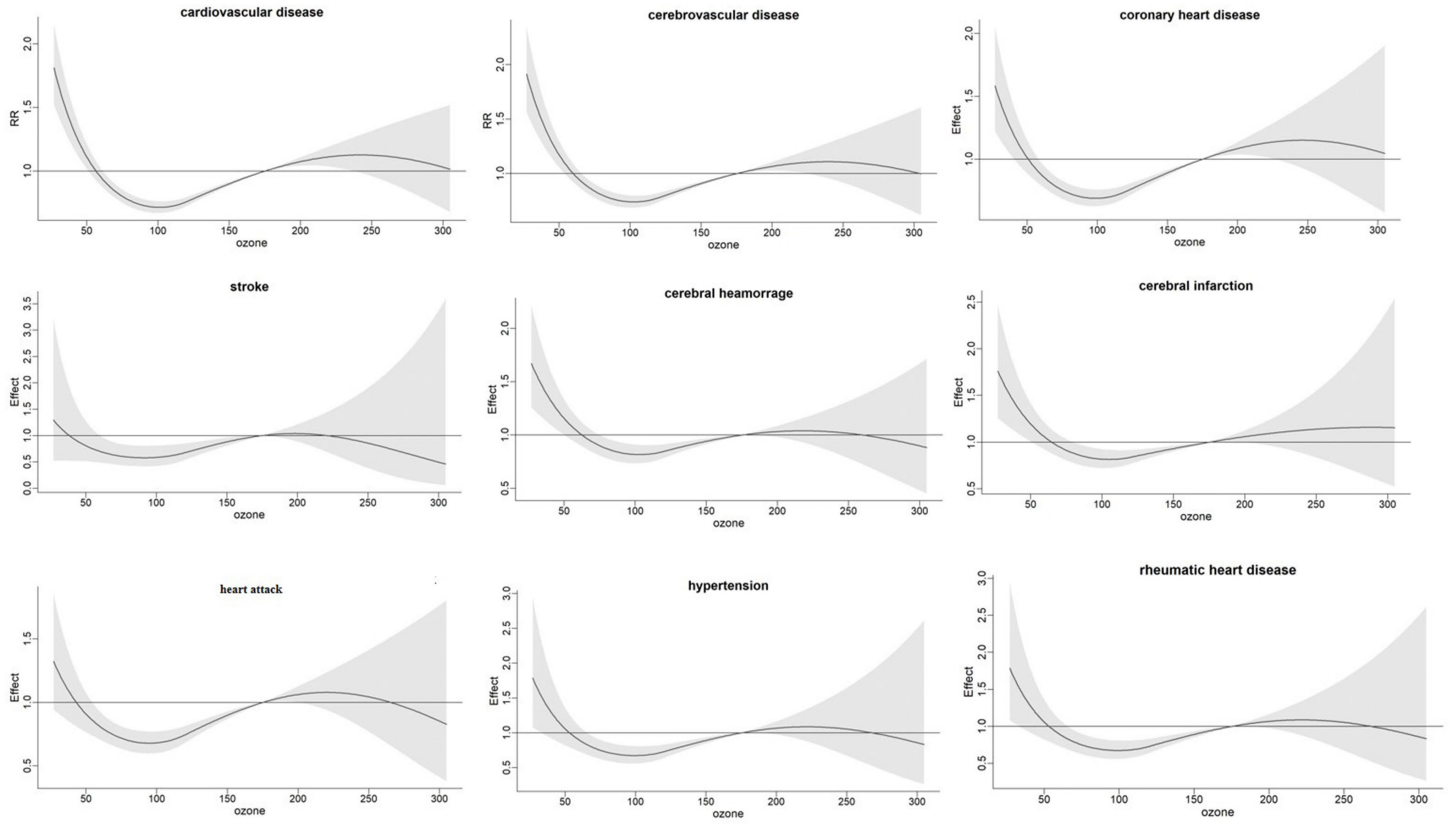
Exposure-response relationship between ozone levels and cumulative relative risk of different types of cardiovascular diseases in Chengdu.

**Figure 2:**
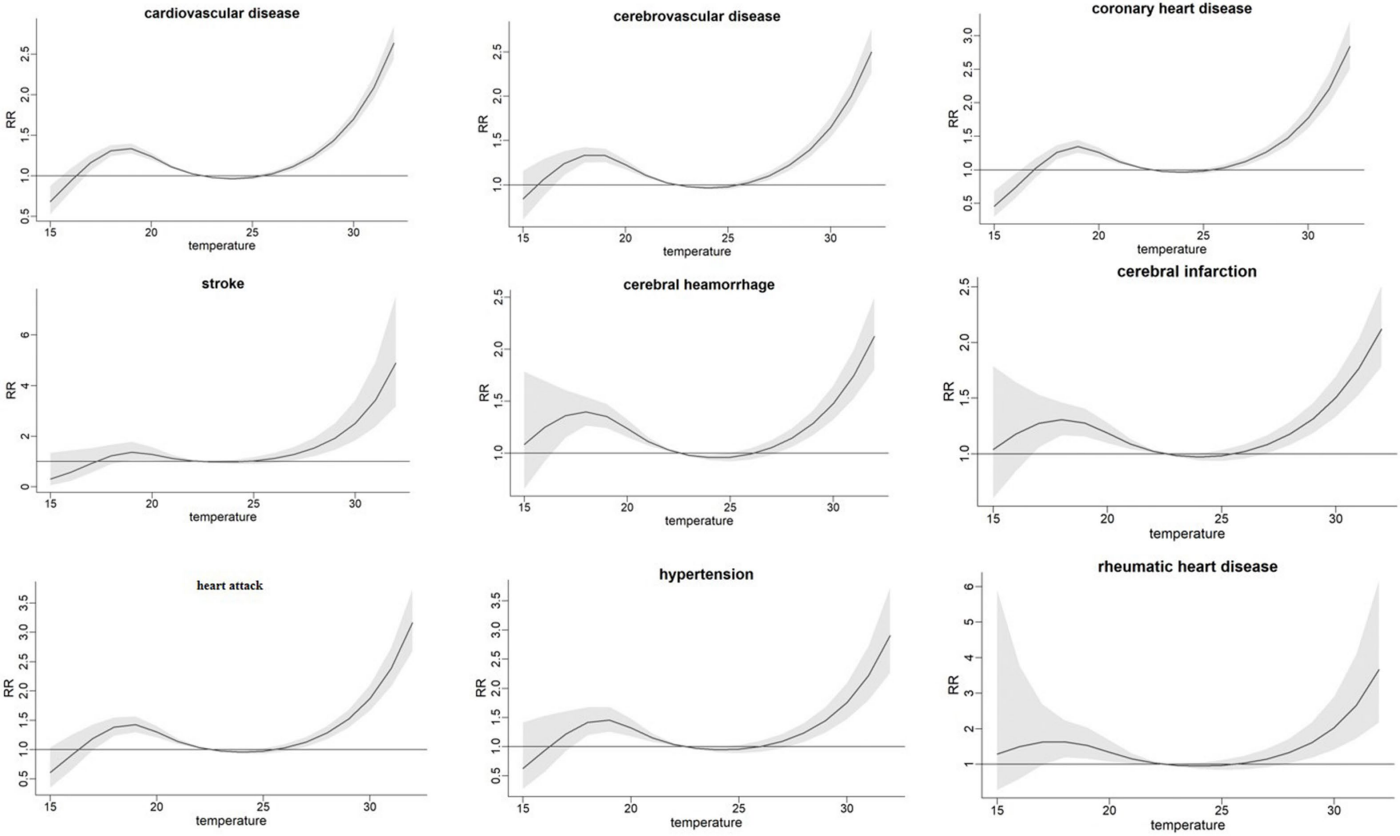
Exposure-response relations between temperature and cumulative relative risk of different types of cardiovascular diseases in Chengdu.

When the ozone concentration was between 150 and 250 ug/m^3^ (Figure 1), and the temperature is higher than 25°C (Figure 2), it shows an increasing trend. Supplementary Figures S2, S3 illustrate the cumulative effects of ozone, high temperature and the death of different types of cardiovascular diseases at lag 0–10. And a “V” shape is observed with cardiovascular disease, cerebrovascular disease, coronary heart disease, cerebral infarction, hypertension and rheumatic heart diseas, a “W” shape with stroke and a “N” shape with cerebral hemorrhage and heart attack in 3D plot (Supplementary Figure S2). The exposure-response curve of temperature on the death of different types of cardiovascular diseases shows “N” shape (Figure 2). And the 3D plot shows the same trend (Supplementary Figure S3).

### 3.4 Effects of high temperature and ozone levels on the death of different types of cardiovascular diseases

**Figure 3:**
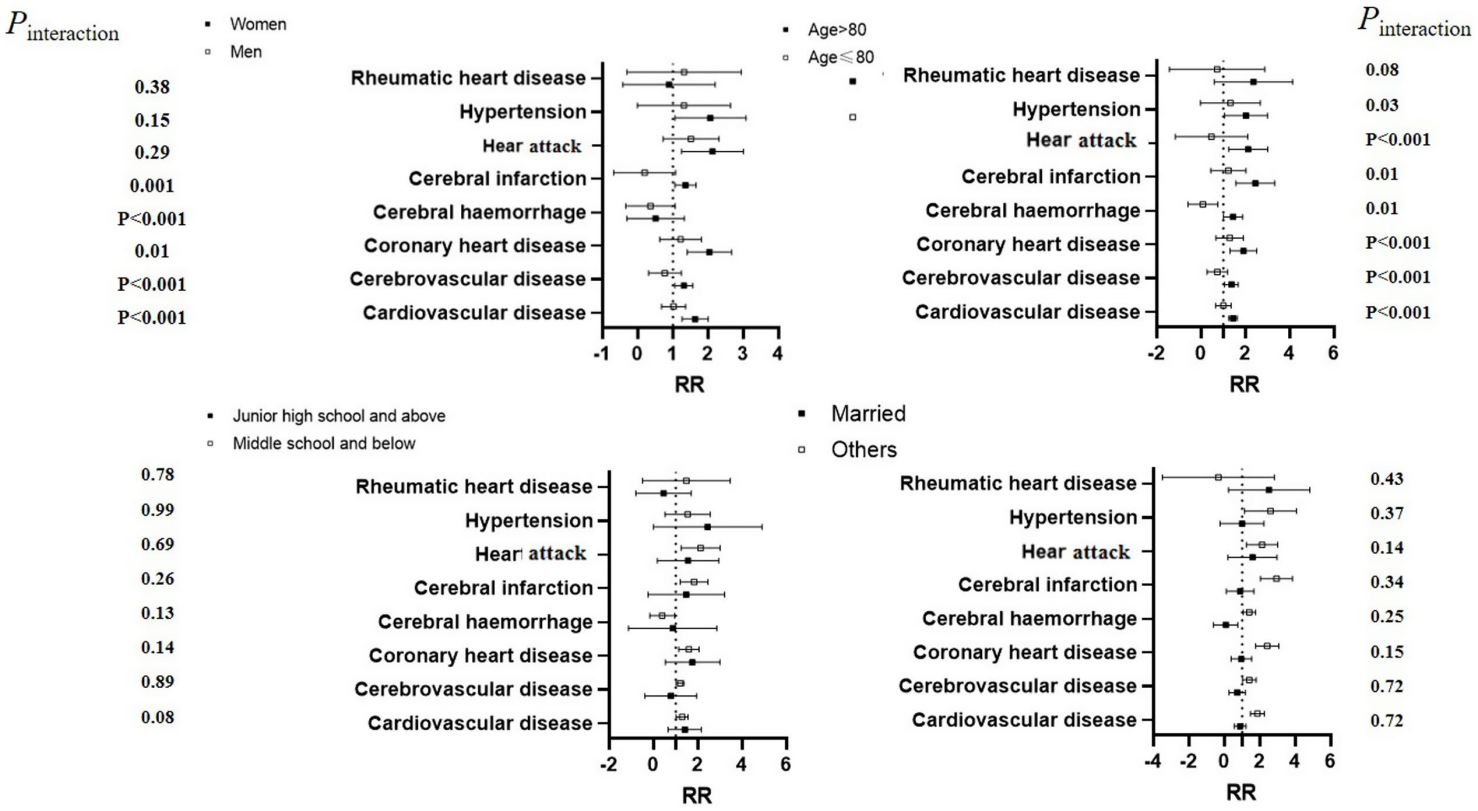
Relative risk of different types of cardiovascular diseases associated with ozone levels, by category of selected risk factors.

We observed positive correlations of ozone levels on the death of cardiovascular disease, cerebrovascular disease, coronary heart disease, cerebral infarction, heart attack, hypertension, and stroke. The risk of death in the ozone-exposed population decreases with increasing single-day lag days. In contrast, the risk of death from cardiovascular diseases in the ozone-exposed population increases with increasing cumulative lag days, and the cumulative lag effect is significantly greater than the single-day lag effect. The highest cumulative lag effect is observed at 3 days (Table 3). The relative risks (RR, 95% CI) were cardiovascular disease (1.26, 1.07–1.54), cerebrovascular disease (1.09, 1.05–1.23), coronary heart disease (1.55, 1.07–2.04), cerebral infarction (1.15, 1.05–1.35), heart attack (1.78, 1.13–2.45), hypertension (1.85, 1.09–2.92), and stroke (1.09, 1.05–1.13), respectively. This effect is more pronounced among women, individuals over 80 years of age, unmarried people, and individuals with lower education (Figure 3).

**Table 3:** Relative risk and 95% confidence interval (95% CI) of relations between ozone levels and the death of different types of cardiovascular diseases.

Variables	lag01	lag02	lag03	lag04	lag05	lag06	lago	lag1	lag2	lag3	lag4	lag5	lag6
Cardiovascular disease	0.65 (0.37, 0.93)	1.02 (0.75, 1.29)	1.26 (1.07, 1.54)	1.43 (1.14, 1.72)	1.59 (1.29, 1.89)	1.68 (1.37, 1.98)	−0.11 (−0.36, 0.13)	0.79 (0.42, 1.17)	0.64 (0.48, 0.81)	0.63 (0.47, 0.79)	0.86 (0.55, 1.18)	0.56 (0.39, 0.72)	0.80 (0.49, 1.11)
Cerebrovascular disease	0.61 (0.23, 0.99)	0.92 (0.44, 1.39)	1.09 (1.05, 1.23)	1.11 (1.06, 1.16)	1.13 (1.02, 1.24)	1.43 (1.21, 1.65)	−0.15 (−0.49, 0.18)	0.66 (0.39, 0.92)	0.45 (0.22, 0.68)	0.36 (0.13, 0.58)	0.39 (0.17, 0.62)	0.43 (0.21, 0.65)	0.43 (0.21, 0.65)
Coronary heart disease	0.64 (0.17, 1.12)	1.16 (0.68, 1.64)	1.55 (1.07, 2.04)	1.79 (1.29, 2.29)	2.12 (1.61, 2.64)	2.26 (1.74, 2.79)	−0.12 (−0.54, 0.29)	0.67 (0.35, 0.99)	0.79 (0.51, 1.08)	0.88 (0.61, 1.17)	0.74 (0.46, 1.02)	0.95 (0.67, 1.23)	0.71 (0.44, 0.98)
Cerebral hemorrhage	0.34 (−0.25, 0.94)	0.47 (−0.11, 1.07)	0.41 (−0.19, 1.01)	0.49 (−0.13, 1.12)	0.44 (−0.19, 1.08)	0.46 (−0.18, 1.12)	−0.32 (−0.84, 0.21)	0.49 (0.09, 0.91)	0.27 (−0.08, 0.63)	0.05 (−0.31, 0.39)	0.23 (−0.12, 0.58)	0.02 (−0.32, 0.36)	0.14 (−0.21, 0.48)
Cerebral infarction	0.59 (−0.11, 1.28)	1.14 (1.05, 1.33)	1.15 (1.05, 1.35)	1.22 (1.07, 1.51)	1.25 (1.26, 1.75)	1.31 (1.32, 1.85)	−0.13 (−0.75, 0.48)	0.63 (0.15, 1.11)	0.28 (−0.14, 0.69)	0.21 (−0.19, 0.62)	0.37 (−0.03, 0.78)	0.54 (0.14, 0.95)	0.36 (−0.03, 0.76)
Heart attack	0.93(0.28, 1.58)	1.37 (0.73, 2.02)	1.78 (1.13, 2.45)	1.93 (1.25, 2.61)	2.12 (1.43, 2.83)	2.22 (1.51, 2.94)	0.02 (−0.56, 0.59)	0.85 (0.41, 1.29)	0.82 (0.44, 1.21)	0.97 (0.59, 1.35)	0.6 (0.28, 1.03)	0.75 (0.38, 1.13)	0.63 (0.26, 1.01)
Hypertension	0.83(−0.21, 1.88)	1.48 (0.44, 2.53)	1.85 (1.09, 2.92)	2.11 (1.02, 3.22)	2.27 (1.14, 3.42)	2.14 (0.99, 3.31)	−0.62 (−1.53, 0.29)	1.15 (0.43, 1.86)	1.01 (0.39, 1.64)	0.95 (0.34, 1.57)	0.84 (0.22, 1.45)	0.72 (0.12, 1.33)	0.21 (−0.39, 0.81)
Stroke	−0.51(−1.23, 0.21)	0.43 (−0.35, 1.21)	1.09 (1.05, 1.13)	1.45 (0.63, 2.29)	2.16 (1.33, 2.99)	2.99 (2.14, 3.85)	−0.55 (−1.14, 0.05)	−0.11 (−0.63, 0.41)	0.87 (0.41, 1.34)	0.65 (0.24, 1.06)	0.79 (0.39, 1.21)	1.38 (0.98, 1.79)	1.78 (1.37, 2.21)
Rheumatic heart disease	0.63 (−1.42, 2.73)	1.07 (−0.99, 3.18)	2.15 (0.02, 4.32)	2.74 (0.53, 4.99)	2.61 (0.34, 4.93)	2.23 (−0.08, 4.59)	−0.46 (−2.28, 1.39)	0.86 (−0.55, 2.29)	0.71 (−0.54, 1.97)	1.76 (0.53, 3.01)	1.41 (0.18, 2.64)	0.34 (−0.87, 1.56)	−0.19 (−1.38, 1.01)

### 3.5 Interactions of high temperature and ozone on the death of different types of cardiovascular diseases

**Figure 4:**
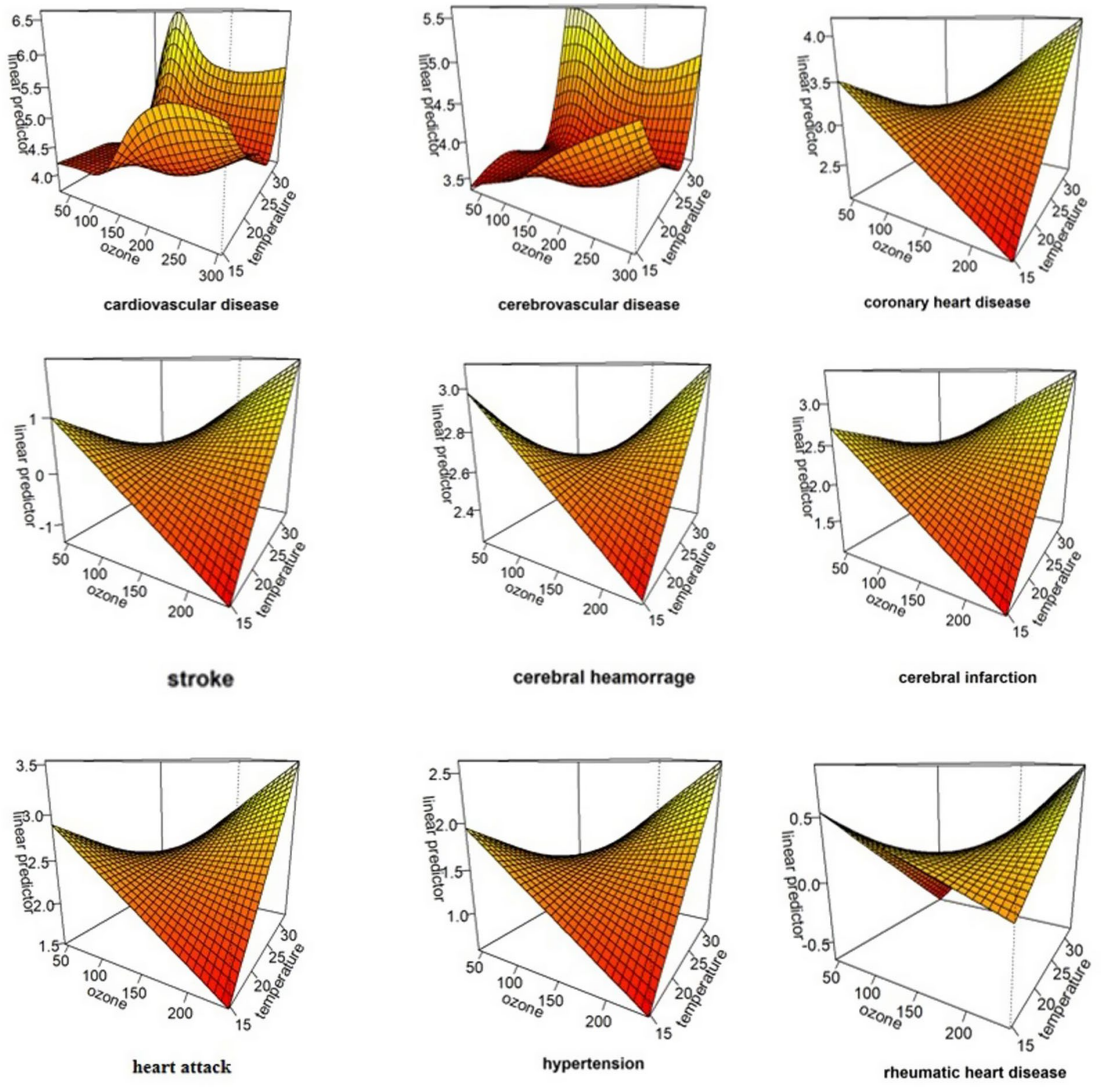
The interaction of temperature and ozone on the morality of different types of cardiovascular diseases.

The results of the qualitative analysis are shown in Figure 4. Under the co-existence of high ozone concentration and high temperature, the risk of mortality from all types of cardiovascular diseases increases. This suggests a potential synergistic effect, whereby higher ozone levels and elevated temperature collectively enhance the risk of death from various cardiovascular conditions.

Further quantitative analysis revealed that high temperature and elevated ozone levels synergistically increased the mortality risk, and the relative excess risk to interaction (RERI, 95% CI) values were cardiovascular disease (0.201, 0.149–0.268), cerebrovascular disease (0.177, 0.099–0.255), coronary heart disease (0.281, 0.169–0.394), cerebral infarction (0.582, 0.369–0.794), stroke (0.287, 0.135–0.441), and 0.482 (0.269–0.694), respectively (Table 4).

**Table 4:** Effects of different interaction items on the incidence of different types of cardiovascular diseases.

Cardiovascular disease	RERI 95% CI	API 95% CI
Cardiovascular disease	0.201 (0.149, 0.268)	0.175 (0.134, 0.216)
Cerebrovascular disease	0.177 (0.099, 0.255)	0.153 (0.096, 0.211)
Coronary heart disease	0.281 (0.169, 0.394)	0.221 (0.151, 0.289)
Cerebral hemorrhage	0.056 (−0.044, 0.155)	0.053 (−0.037, 0.143)
Cerebral infarction	0.582 (0.369, 0.794)	0.373 (0.288, 0.459)
Heart attack	0.287 (0.135, 0.441)	0.223 (0.131, 0.316)
Hypertension	0.174 (−0.031, 0.379)	0.156 (−0.001, 0.312)
Stroke	0.482 (0.269, 0.694)	0.173 (0.188, 0.359)
Rheumatic heart disease	0.435 (−0.048, 0.917)	0.295 (0.062, 0.529)

## 4 Discussion

We observed nonlinear associations between daily mean temperature and ozone levels on the number of deaths from different types of cardiovascular diseases, with the highest effect occurring at a cumulative lag of 3 days. Positive interactions between ozone levels and high temperature were noted in relation to deaths from cardiovascular disease, coronary heart disease, heart attack, hypertension, stroke and rheumatic heart disease. These associations were more pronounced among women, the older adult, individuals with lower education levels, and unmarried people.

In our analysis, the average ozone level from May to September during the summer was 126.69 μg·m^−3^. Based on hourly ozone monitoring data from 1,836 monitoring stations across 338 cities in China in 2019, the average of ozone concentration in summer is 109 μg/m^3^ (24). In the summer of 2017, surface ozone levels exceeded 200 μg/m^3^ in major cities throughout China (25). According to GB3095-2012, the limits for O_3_-1h and O_3_-8h are set at 200 and 160 μg/m^3^, respectively (26). The WHO reference limit for ozone is 60 μg/m^3^ (27). Our results indicate that the current standard for ozone hazard limit for cardiovascular disease still lies some limitations, so further adjustments to national air quality standards are necessary.

At present, there is no consensus on the interaction between ozone and high temperature and cardiovascular deaths. There may be some possible influencing factors: (1) The economic development status and the population characteristics of different cities are different, this will affect the levels of its air pollution, thus inducing different health effects. (2) The methodology used varies, studies carried in Shijiazhuang and Shenzhen (14, 16) used the GAM model to explore the synergistic effect of high temperature and ozone on the number of deaths from circulatory system diseases, while the GAM model lacks structured lag modeling. Study (19) carried in 95 large US communities used a nonparametric regression model to explore interactive effects of temperature and ozone on cardiovascular mortality, which may also ignore the effects of lag. (3) Different regions have their own climate and geographical location differences, which makes the research conclusion different.

Our analysis revealed nonlinear exposure-response patterns for temperature-ozone interactions in cerebrovascular disease, cerebral hemorrhage, and acute myocardial infarction mortality, characterized by N-shape curves with inflection points at 25°C (temperature) and 100 μg/m^3^ (ozone). This exposure-response curve is consistent with the findings of Rui Pan (28). Additionally, a study conducted on a US Medicare cohort also found that long-term ozone exposure is associated with cardiovascular disease morality, with a RR of 1.005 (95% CI: 1.003, 1.007) (21). Of note, previous epidemiological evidence has primarily explored associations between ozone and overall or specific cardiovascular disease, while our study focuses for the first time on multiple types of cardiovascular diseases.

In our study, we observed the interactions of ozone levels and high temperature on the death of cardiovascular disease, cerebrovascular disease, coronary heart disease, cerebral infarction, stroke and heart attack. Ozone is formed by the chemical reaction of precursor chemicals such as nitrogen oxides and organic chemicals with sunlight, and relations between ozone concentrations and temperature have been fully explored. Higher temperature exacerbate the chemical reactions associated with ozone production and also increasing emissions of precursor chemicals, leading to higher ozone concentrations (29). The temperature-dependent environmental chemical mechanism of ozone generation hints at potential interactions between temperature, ozone, and public health (30). Nevertheless, the biological mechanisms behind the association between temperature, ozone, and cardiovascular disease are not yet fully understood. Animal experiments have indicated that simultaneous exposure to ozone and high temperature increases inflammatory factors, thrombogenic factors, activation of fibrinolytic and oxidative stress pathways, which may be potential physiological mechanisms contributing to the worsening of cardiovascular disease (25). However, currently no human evidence exists to suggest that the interaction of ozone and high temperatures leads to inflammation and thrombosis, and more epidemiologic evidence is required to more closely investigate the mechanisms of environmentally related cardiovascular disease development in the future (31).

Stratified analysis revealed that women, the older adult, individuals with lower education, and unmarried people are more vulnerable to changes in the ozone levels. This gender disparity aligns with global mortality patterns where cardiovascular disease predominates in female fatalities (32). Studies have indicated that women are more sensitive to fluctuations in ozone, with hormonal differences likely being a primary factor (33). Estrogens, which are lipophilic steroid hormones primarily produced in the ovaries, regulate female differentiation and influence vascular function, the inflammatory response, and metabolism, thereby affecting cardiovascular risk factors (34). The older adult are more susceptible to chronic diseases (35), which are more sensitive to changes in ozone and temperature changes. This finding is consistent with previous studies (36, 37). Furthermore, research suggests that varying environmental exposure may contribute to health vulnerabilities (38). Different levels of ozone exposure are influenced by activity patterns (39, 40), the older adult tend to have more time to outdoor activities, potentially leading to higher cumulative ozone exposure. Additionally, individuals with lower education levels are likely to encounter greater ozone pollution in their work environments and for extended periods.

We found that the associations were more pronounced in individuals with lower education levels, which is consistent with previous studies (41, 42). Education is a significant social determinant of health, and its attainment is linked to various health outcomes (43). Higher educational attainment is associated with health-related behaviors that may contribute to the risk of cardiovascular disease. Educated individuals may have greater access to health care and engage in healthier behaviors (44). In addition, well-educated people may obtain and afford health treatment (45). Additionally, another important finding is that marital status also influences the associations. Marital status was divided into two categories: married and others (widowed, divorced and never married). We found that married individuals had a lower morality risk of developing cardiovascular disease. This is consistent with the study conducted by Devinder Singh Dhindsa (46), which showed that married people experience a decreased rate of adverse cardiovascular events. Numerous studies have reported that marital status significantly affects people’s health and mortality, the pressure for unmarried individuals are mainly from the society (46–48). Marriage has a protective effect, married individuals can receive greater emotional support from their spouses, which makes them more resilient to risk from the society (49). Given the dual challenges of escalating cardiovascular diseases among women, the older adult, individuals with lower education levels, future research should continue to identify risk factors.

Our study has several strengths. Firstly, it is a 10-year study that includes more than 352 thousand deaths due to cardiovascular disease. Secondly, we used the DLNM model to further explore the lagged effects of ozone exposure and high temperature. Thirdly, to our knowledge, this is the largest and most recent study examining the interaction of co-exposure to ozone and high temperature in the central Sichuan basin of southwest China. However, there are still some limitations to our study. Firstly, our meteorological and environmental data were extracted from fixed Environmental Monitoring sites, which may not reflect the true exposure levels of individuals. Secondly, although the time series analysis controlled for some time-related confounding factors, the influence of economic status and medical conditions could not be quantified in the model due to limitations in the research methodology and the data collected. Additionally, baseline health status factors (smoking, obesity, occupation, etc) were not considered. Thirdly, the study was limited to single city, making it difficult to generalize our results. Fourthly, we acknowledged the limitation of our study’s short-term exposure assessment. Further large-scale prospective cohort studies on long-term effects including multiple cities are necessary for future analysis.

## 5 Conclusion

We observed the synergistic interactions between ozone levels and high temperature on the death of cardiovascular disease, cerebrovascular disease, coronary heart disease, cerebral infarction, heart attack and stroke. The associations were stronger in women, the older adult, individuals with lower education levels, and unmarried people. Given the dual challenges of escalating cardiovascular diseases and persistent ozone pollution, targeted intervention strategies for high-risk populations are urgently needed.

## Data Availability

The data analyzed in this study is subject to the following licenses/restrictions: our data are from Chengdu Meteorological Bureau, Chengdu Environmental Monitoring Centre, the Death Surveillance System of China Center for Disease Control and Prevention separately. Requests to access these datasets should be directed to Chengdu Meteorological Bureau, Chengdu Environmental Monitoring Centre, the Death Surveillance System of China Center for Disease Control and Prevention.
