# Supplementary material for: Interaction effects of high temperature and ozone on cardiovascular disease mortality in Chengdu, 2014–2023
Source: Front Public Health. 2025 Jul 10;13:1580849. doi: 10.3389/fpubh.2025.1580849 (PMC12287009; doi:10.3389/fpubh.2025.1580849)
Supplement: Supplementary file 1 [file Data_Sheet_1.docx]

**Supplementary material**

**The interaction effects of high temperature, ozone on the death of different types of cardiovascular diseases in Chengdu, 2014-2023**

Jinqiu Yao^a^, Jingwen Sun^a^, Dan Kuang^a^, Wen Qian^a^, Li Luo^a^, Fangkui Qin^a^, Yifan Zhai^a^, Yueling Li^a^, Jiaqi Huang^a^, Cheng Wang^a,*^, Rong Lu^a,*^, XuFang Gao^a,*^

^a^Chengdu Center for Disease Control and Prevention, Chengdu, China

**Supplementary Figure S1.** Temperature and ozone concentrations from 2013 to 2023 in Chengdu

**Supplementary Table S1.** Spearman’s correlation coefficients between meteorological factors and environmental data

**Supplementary Figure S2.** The 3D plots of ozone levels on the death of different types of cardiovascular diseases at 0-10 lag days

**Supplementary Figure S3.** The 3D plots of temperature on the death of different types of cardiovascular diseases at 0-10 lag days

**Supplementary Figure S4.** The QAICs of the fitted model of ozone effects for choosing various dfs of the ns controlling the long-term trends and seasonality

**Supplementary Figure S5.** The QAICs of the fitted model of ozone effects for choosing various dfs of the ns for the exposure-response association

**Supplementary Figure S6.** The QAICs of the fitted model of ozone effects for choosing various dfs of the ns for the lag-response association

**Supplementary Figure S7.** Distribution of 14 environmental protection

state-controlled sites in Chengdu

**
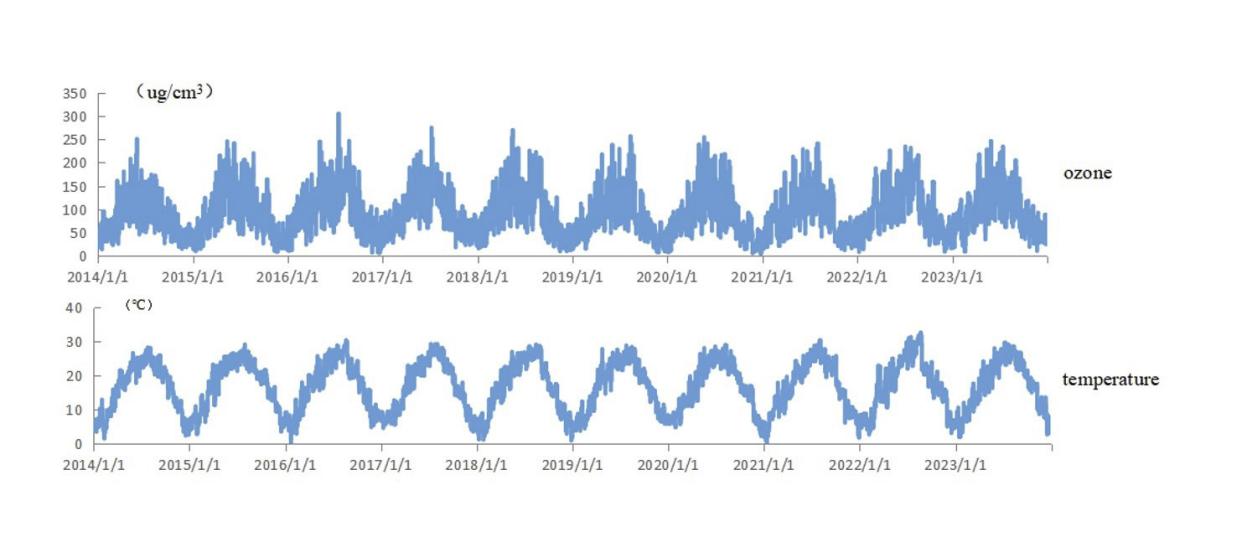
**

**Supplementary Figure S1.** Temperature and ozone concentrations from 2013 to 2023 in Chengdu

**Supplementary Table S1.** Spearman’s correlation coefficients between meteorological factors and environmental data

| Variables | PM_25_ | NO_2_ | SO_2_ | CO | O_3_ | pressure | temperature | humidity |
| --- | --- | --- | --- | --- | --- | --- | --- | --- |
| PM_25_ | 1 | 0.59** | 0.61* | 0.63* | -0.31* | 0.06 | -0.25* | -0.13* |
| NO_2_ | 0.59* | 1 | 0.51* | 0.69* | 0.28* | 0.04 | -0.21* | -0.24* |
| SO_2_ | 0.61* | 0.51* | 1 | 0.63* | 0.15* | 0.07 | -0.19* | -0.15* |
| CO | 0.65* | 0.69* | 0.63* | 1 | 0.27 | -.17* | 0.04 | 0.09* |
| O_3_ | -0.31* | 0.28* | 0.15* | 0.27 | 1 | -.31* | 0.32* | -0.39** |
| pressure | 0.06 | 0.04 | 0.07 | -0.17* | -0.31* | 1 | -0.68* | -0.07* |
| temperature | -.25* | -0.21* | -0.19* | 0.04 | 0.32* | -.68* | 1 | 0.03 |
| humidity | -0.12* | -0.24* | -0.15* | 0.09* | -0.39** | -.07* | 0.03 | 1 |

**Note:***, *P*<0.05; **, *P*<0.01


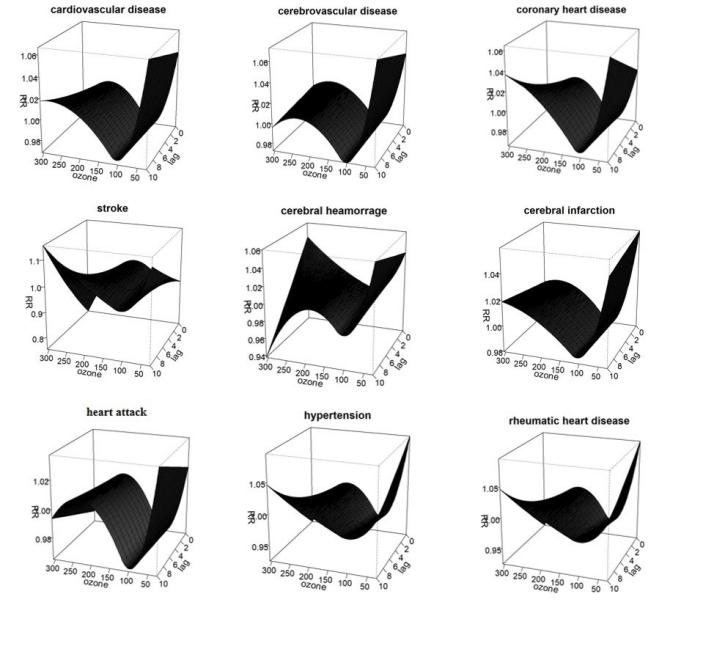


**Supplementary Figure S2.** The 3D plots of ozone levels on the death of different types of cardiovascular diseases at 0-10 lag days


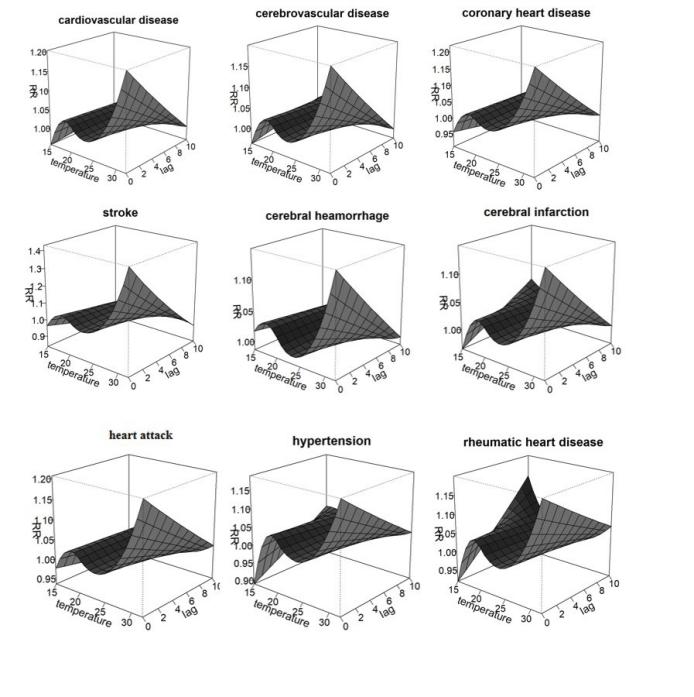


**Supplementary Figure S3.** The 3D plots of temperature on the death of different types of cardiovascular diseases at 0-10 lag days

**Sensitivity analysis**

(Take cardiovascular disease as an example)

1. To control the long-term trends and seasonality in the time series, we conducted a sensitivity analysis to change the df of the natural cubic spline (ns) from 1 to 10 per year. As shown in **Supplementary Figure S4**. the ns with 7df per year is reasonable to control the long-term trends and seasonality in the time series.


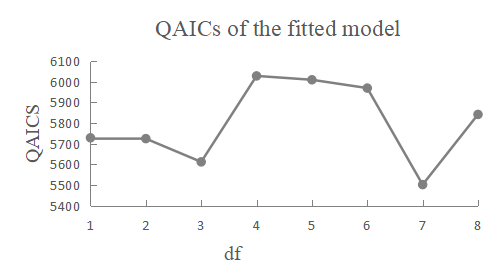


**Supplementary Figure S4.** The QAICs of the fitted model of ozone effects for choosing various dfs of the ns controlling the long-term trends and seasonality

1. The choice of the df defining the exposure-response association of ozone and cardiovascular disease

A sensitivity analysis was conducted to determine the df of the ns for the exposure-response relationship between ozone and cardiovascular disease by varying the df from 2 to 8. As shown in **Supplementary Figure S5**, the ns with 3df exhibited the smallest QAIC, indicating the goodness of fit was the best. Therefore, we chose a ns with 3df to fully capture the exposure-response association of ozone and cardiovascular disease.


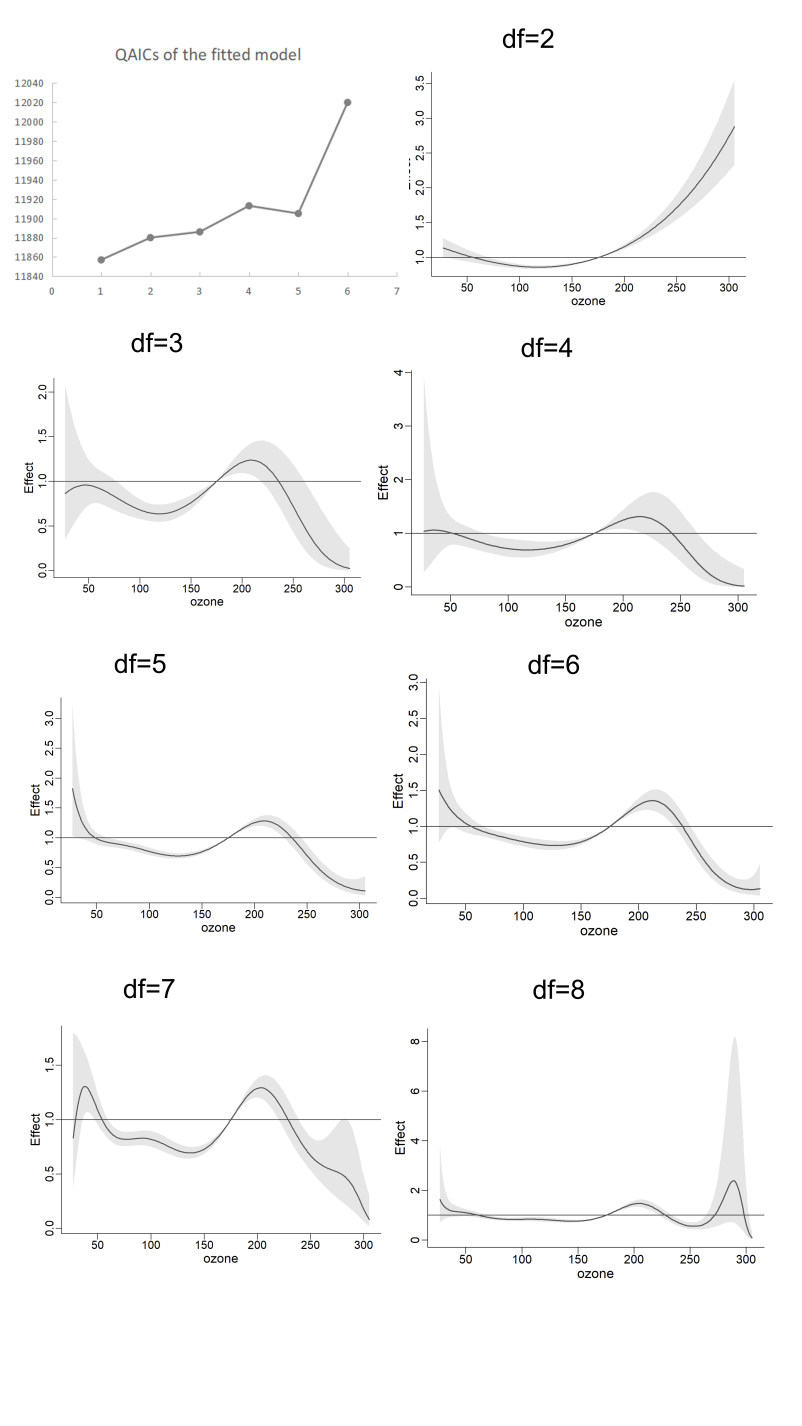


**Supplementary Figure S5.** The QAICs of the fitted model of ozone effects for choosing various dfs of the ns for the exposure-response association

1. To explore the lag structure of the effects of ozone on cardiovascular disease, we carried out a sensitivity analysis with lag days up to 30 days. Based on the results shown in **Supplementary Figure S6**, we chose a lag range of 0-10 days to fully capture the lagged effect of ozone on cardiovascular disease. **.**

**
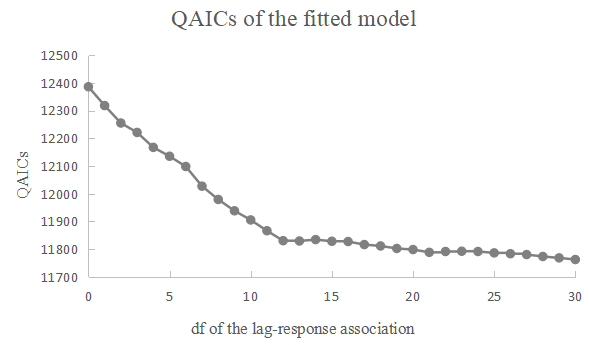
**

**Supplementary Figure S6.** The QAICs of the fitted model of ozone effects for choosing various dfs of the ns for the lag-response association


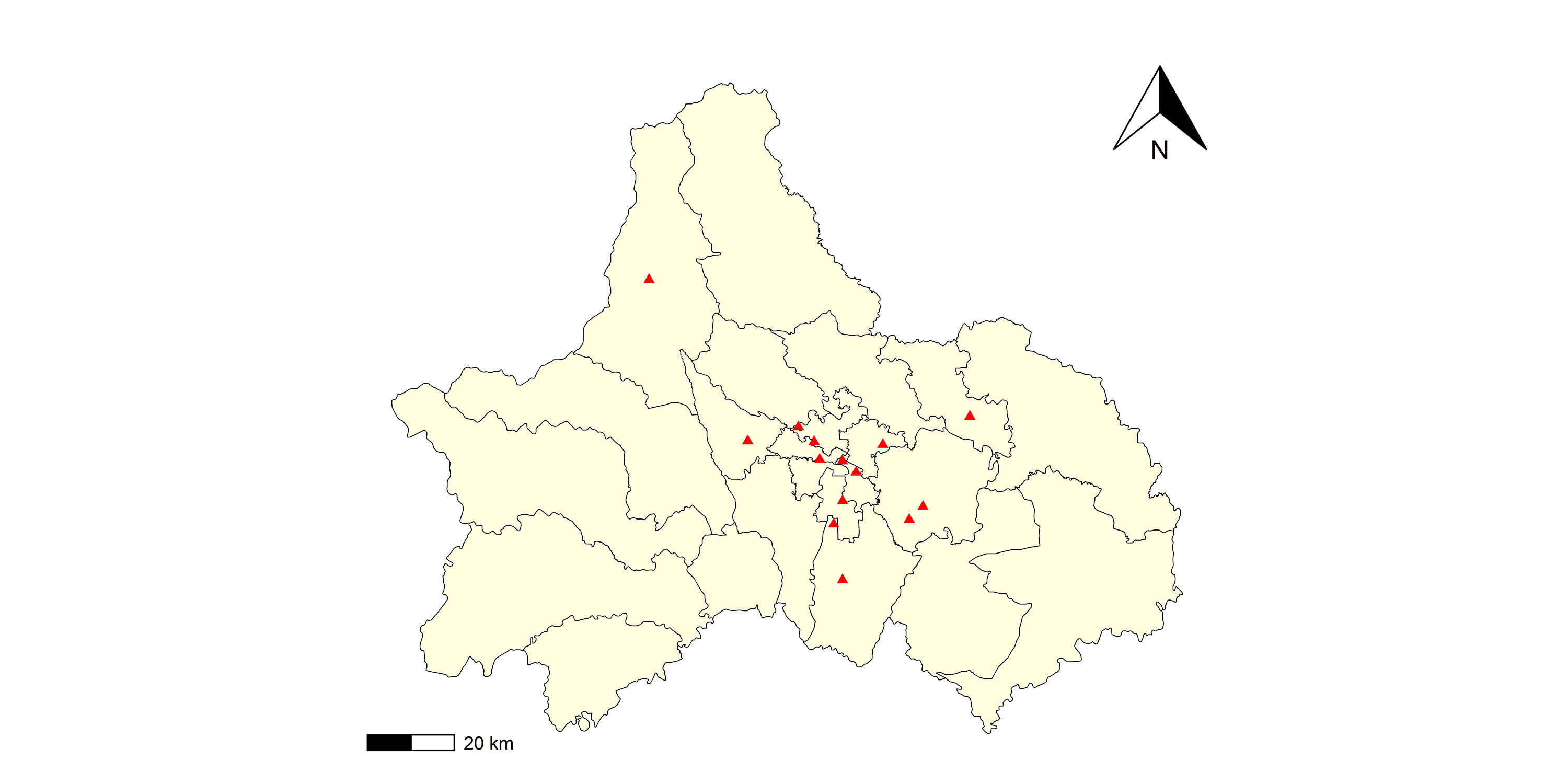


Supplementary Figure S7. Distribution of 14 environmental protection

state-controlled sites in Chengdu
